# Effects of Intra-Articular Administration of High-Molecular-Weight Linear Hyaluronic Acid on Synovial Fluid Characteristics and Joint Environment

**DOI:** 10.3390/antiox15081041

**Published:** 2026-08-21

**Authors:** Marcela dos Santos Ribeiro, Vittoria Guerra Altheman, Victória Ferreira Alexandre, Anna Paula Balesdent Barreira, Lorena Cardozo Ferrari, Letícia de Oliveira Cota, Paulina Betancur Guerra, Emanuel Vitor Pereira Apolonio, Daniel L. Parra-Torres, Marcos Jun Watanabe, Heitor Cestari, Fabiana Ferreira de Souza, Raquel Yvonne Arantes Baccarin, Ana Liz Garcia Alves

**Affiliations:** 1 Department of Surgery and Animal Reproduction, School of Veterinary Medicine and Animal Science, São Paulo State University (FMVZ/UNESP), Botucatu 18618-618, SP, Brazil; marcela.s.ribeiro@unesp.br (M.d.S.R.);; 2 Department of Veterinary Medicine and Surgery, Institute of Veterinary Medicine, Federal Rural University of Rio de Janeiro (UFRRJ), Seropédica 23890-000, RJ, Brazil; 3 Department of Internal Medicine, School of Veterinary Medicine and Animal Science (FMVZ/USP), São Paulo 05508-270, SP, Brazil

**Keywords:** synovitis, equine, oxidative stress, lipid peroxidation, TBARS, redox

## Abstract

Synovitis is an inflammatory disorder in horses that contributes to cartilage degradation and osteoarthritis. This study evaluated the effects of intra-articular administration of high-molecular-weight (HMW), linear (non-cross-linked) hyaluronic acid (HA) in an equine model of lipopolysaccharide (LPS)-induced acute synovitis. In this randomized blinded study, acute synovitis was induced in radiocarpal joints of 16 adult horses using LPS (0.25 ng). After 12 h, joints were treated intra-articularly with either 2 mL of phosphate-buffered saline (PBS; control group) or 2 mL of HMW-HA (20 mg/mL; 2280 kDa; treatment group). Horses underwent orthopedic, ultrasonographic, and synovial fluid evaluations. Horses treated with HMW-HA exhibited lower synovial membrane thickening and reduced lameness scores compared with controls. Synovial fluid analysis demonstrated increased concentrations of HMW-HA during the acute inflammatory phase in treated joints. Reduced chondroitin sulfate release was also detected following HMW-HA administration. Lower lipid peroxidation levels (TBARS) were observed in the treated group at 24 h and 14 days. Intra-articular administration of high-molecular-weight linear hyaluronic acid was associated with increased synovial availability of HMW-HA during the acute inflammatory phase, attenuation of pain-related clinical signs and synovial membrane thickening, and modulation of the redox environment. These findings support a modulatory role of linear HMW-HA in the intra-articular environment.

## 1. Introduction

Hyaluronic acid (HA) is a non-sulfated, unbranched glycosaminoglycan that is an essential component of the extracellular matrix and synovial fluid. Its structure consists of repeating disaccharide units of β1–3 D-N-acetylglucosamine and β1–4 D-glucuronic acid. Under normal physiological conditions, HA exhibits a high molecular weight and hydrophilic properties, which confer viscoelasticity and water-retention capacity [1,2,3,4].

The molecular size of hyaluronic acid (HA) directly influences its water-binding capacity: the greater the molecular weight, the greater the viscoelastic and shock-absorbing properties of high-molecular-weight HA (HMW-HA). Conversely, low-molecular-weight HA (LMW-HA), owing to its reduced water-binding capacity, exhibits diminished viscoelastic characteristics, compromising the shock-absorbing function of synovial fluid [5,6].

In addition to its structural role, HA acts as a signaling molecule through its interactions with receptors, such as CD44. Although this binding occurs regardless of molecular size, the biological effects differ: low-molecular-weight and oligomeric HA activate proinflammatory pathways, whereas high-molecular-weight HA exerts anti-inflammatory, anabolic, and analgesic effects [3,7,8,9]. In vitro studies have shown that HA stimulates stem cell proliferation and differentiation into chondrocytes, as well as the synthesis of extracellular matrix components, including collagen, aggrecan, and endogenous HA by mature chondrocytes. Moreover, HA modulates macrophage polarization, promoting anti-inflammatory phenotypes [1,7,8,10].

Based on these biological and physicochemical properties, intra-articular HA formulations can be broadly classified into linear and chemically reticulated forms. Linear HA formulations are structurally closer to endogenous HA and act primarily through cellular signaling mechanisms, interacting with chondrocytes and synoviocytes and contributing to the maintenance of articular homeostasis [11].

In contrast, reticulated formulations involve chemical cross-linking between polymer chains, resulting in increased molecular stability, prolonged intra-articular residence time, and the formation of a three-dimensional gel-like network [11]. Reticulated HA exhibits a predominantly mechanical mode of action, with viscoelastic rheological properties that enhance shock absorption and joint cushioning [11].

Considering the distinct biological behavior of linear hyaluronic acid, the present study focuses on the intra-articular administration of a high-molecular-weight, non-cross-linked HA formulation, with emphasis on its biological and modulatory effects within the articular environment.

During synovitis, the inflamed synovial membrane releases cytokines, including interleukins 1, 6, and 10, tumor necrosis factor (TNF), and enzymes such as hyaluronidases, which contribute to the degradation of endogenous HA. Fragmentation of high-molecular-weight HA into smaller molecules exacerbates inflammation, perpetuating synovial damage. The resulting decrease in synovial fluid viscosity, combined with increased matrix metalloproteinase (MMP) activity, accelerates cartilage degeneration and promotes osteoarthritis development [2,3,12,13,14].

In addition to inflammatory mediators, oxidative processes also play an important role in the progression of synovial alterations. Increased production of reactive oxygen and nitrogen species during synovitis contributes to cellular damage, extracellular matrix degradation, and imbalance of the joint microenvironment. These changes may persist beyond the acute inflammatory phase, reinforcing the importance of redox balance in the maintenance of joint homeostasis [15,16].

Interrupting this inflammatory cycle and restoring the rheological properties of synovial fluid are essential for preserving cartilage and joint integrity. Although anti-inflammatory drugs provide symptomatic relief, they lack regenerative capacity and may compromise long-term joint health in athletes. HA-based therapies are increasingly employed in both human and veterinary medicine to manage synovitis and joint dysfunction, with promising clinical outcomes [9,12,17].

The objective of this study was to evaluate the effects of intra-articular administration of high-molecular-weight linear hyaluronic acid on synovial fluid characteristics and joint environment through physical, cellular, biochemical, orthopedic, and ultrasonographic assessments.

## 2. Materials and Methods

### 2.1. Ethics Committee Approval

This study was approved by the Animal Use Ethics Committee (CEUA) of the Faculty of Veterinary Medicine and Animal Science (FMVZ), São Paulo State University (UNESP), registered at the National Council for Animal Experimentation Control (CONCEA) under CIAEP no. 01.0115.2014. The experimental protocol was approved under CEUA no. 0172/2022, approved on 12 August 2022, and conducted in accordance with Brazilian legislation (Law no. 11.794/2008; Decree no. 6.899/2009) and CONCEA guidelines.

### 2.2. Experimental Design

A randomized, blinded experimental study was conducted at the Large Animal Surgery Unit and associated laboratories of FMVZ/UNESP, Botucatu, Brazil. Sixteen clinically healthy adult horses (>12 months old) of mixed breed and both sexes were included in the study.

**Figure 1:**
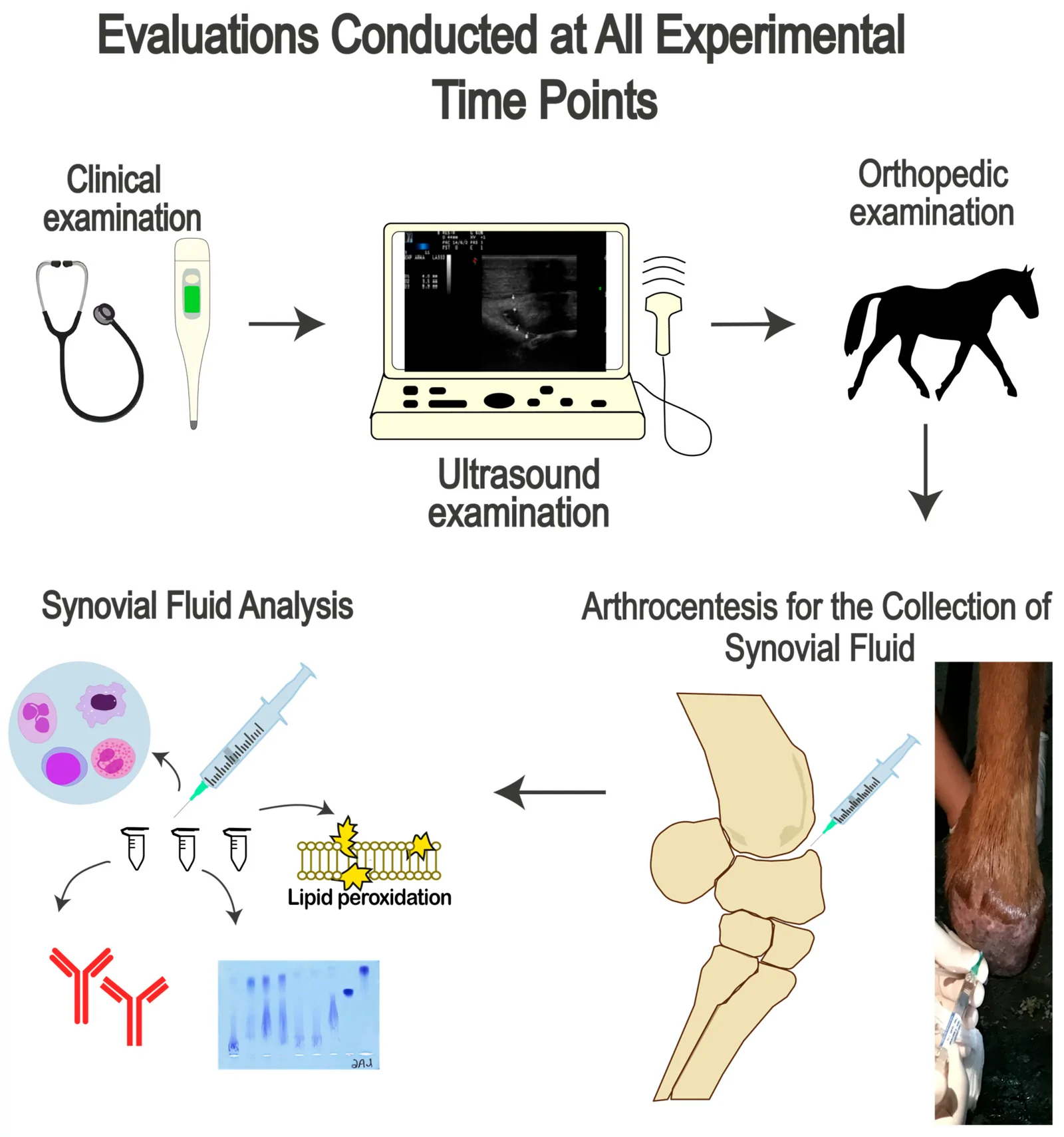
Diagram illustrating the study design and evaluations conducted at each time point. All horses underwent physical, orthopedic, and ultrasound examinations. Synovial fluid was collected by arthrocentesis for biochemical, cytological, enzyme-linked immunosorbent assay (ELISA), and electrophoretic analyses.

Before enrollment (D − 2), all animals underwent clinical, orthopedic, radiographic, hematological, and biochemical evaluations to confirm the absence of joint disease or systemic illness (Figure 1). Horses were eligible if they showed no history or clinical signs of lameness, synovitis, or osteoarthritis and were not receiving any medication at the time of the study.

Animals were randomly allocated into two groups with homogeneous distribution of age and body weight: a control group (n = 8) and a treatment group (n = 8). Randomization was performed by a member of the research team not involved in outcome assessments.

All clinical, ultrasonographic, and laboratory evaluations were performed by blinded assessors. The individual responsible for treatment administration did not participate in subsequent evaluations.

Horses were housed under a semi-intensive system in collective paddocks (25,000 m^2^; ≥100 m^2^ per animal), fed 2 kg/day of concentrate and Tifton hay, had access to Coast Cross pasture, and received water ad libitum.

The control group (n = 8) consisted of 4 geldings and 4 mares, whereas the treatment group (n = 8) included 6 geldings and 2 mares. Groups were balanced regarding age and body weight.

### 2.3. Synovitis Induction and Treatment

Acute synovitis was induced in one randomly selected radiocarpal joint of each horse via arthrocentesis of the dorsal radiocarpal recess, followed by intra-articular injection of 2 mL of sterile phosphate-buffered saline (PBS, pH 7.4; Gibco, Thermo Fisher Scientific, Grand Island, NY, USA) containing 0.25 ng of lipopolysaccharide (LPS) from Escherichia coli serotype O55:B5 (Sigma-Aldrich^®^, St. Louis, MO, USA). The experimental model was based on previously validated equine models of acute synovitis induced by intra-articular administration of LPS, which have been used to reproduce the clinical, cytological, biochemical, and inflammatory changes observed during acute synovial inflammation in horses [18,19,20,21].

Twelve hours (12 h) after synovitis induction, animals underwent clinical, orthopedic, and imaging evaluations, followed by synovial fluid collection. Immediately after these assessments, joints received intra-articular treatment. The treatment group received 2 mL of high-molecular-weight linear (non-cross-linked) hyaluronic acid (20 mg/mL; 2280 kDa; Pró-joint^®^, Botupharma^®^, Botucatu, SP, Brazil), derived from bacterial fermentation (non-animal origin). This dose corresponded to the manufacturer’s recommended intra-articular therapeutic dose. Control joints received an equivalent volume (2 mL) of phosphate-buffered saline under the same conditions.

### 2.4. Orthopedic Evaluation

Orthopedic assessments were performed at baseline (0 h) and at 12 h, 24 h, 48 h, 7 days, and 14 days post-induction. Evaluations included joint effusion and lameness assessment at walk and trot, as well as after flexion, following standardized equine orthopedic examination guidelines recommended by the American Association of Equine Practitioners (AAEP).

### 2.5. Ultrasonography

Ultrasonographic examinations were performed at all experimental time points using an Esaote MyLab70 ultrasound system (Esaote S.p.A., Genoa, Italy) equipped with an 8–17 MHz linear transducer. Longitudinal images of the radiocarpal joint were obtained in triplicate between the extensor carpi radialis and common digital extensor tendons. Synovial membrane thickness was measured at a standardized anatomical reference point on the distal radius epiphysis, 4 mm distal to the joint space. Mean values from triplicate measurements were used for quantitative analysis.

### 2.6. Arthrocentesis

Synovial fluid was collected by arthrocentesis under aseptic conditions at all experimental time points using a 30 × 0.8 mm hypodermic needle and a 5 mL syringe. Samples were obtained immediately before synovitis induction and at subsequent evaluations. At 0 h, horses were sedated with xylazine hydrochloride (10%, 1 mg/kg IV) [22]; subsequent collections used physical restraint and limb flexion only. Synovial fluid was aliquoted into vacuum tubes with and without EDTA to allow cytological, physical, and biochemical analyses. Samples intended for physicochemical analyses were stored at 4 °C, whereas samples designated for ELISA, electrophoresis, and TBARS analyses were stored at −80 °C. Due to limited sample volume, not all analyses could be performed at all time points for all animals. Consequently, sample size varies for specific analyses, particularly electrophoresis and TBARS, depending on sample availability.

### 2.7. Synovial Fluid Analysis

Synovial fluid was evaluated for physical (volume, clotting, color, appearance), chemical (pH, glucose, occult blood), and cellular (erythrocytes, neutrophils, mononuclear cells) characteristics. pH, glucose, and occult blood were assessed using commercial reagent strips; density, protein, and fibrinogen were determined via refractometry.

### 2.8. ELISA

The cartilage degradation biomarker C2C was quantified using commercial ELISA kits (C2C: cat#60-1001-001, IBEX Pharmaceuticals Inc., Montréal, QC, Canada) following manufacturer protocols.

### 2.9. Lipid Peroxidation

Lipid peroxidation in synovial fluid was assessed using the thiobarbituric acid reactive substances (TBARS) assay. Briefly, synovial fluid samples were mixed with 10% trichloroacetic acid for protein precipitation and centrifuged to obtain the supernatant. Subsequently, samples were incubated with thiobarbituric acid (TBA) solution and heated at 90–100 °C for 15 min to allow the reaction between TBA and malondialdehyde (MDA).

After cooling, the absorbance of the resulting chromogen was measured spectrophotometrically at 532 nm. Lipid peroxidation levels were determined by comparison with a standard curve constructed using known concentrations of MDA and expressed as MDA equivalents (ng/mL).

### 2.10. Electrophoresis of Synovial Fluid

Electrophoresis was performed to quantify the HA concentration and molecular weight distribution following Neuenschwander et al. [18]. The samples were digested with alkaline protease, denatured in boiling water, centrifuged, vacuum-dried, rehydrated, and subjected to agarose gel electrophoresis in PDA buffer (0.05 M, pH 9.0). HA was fixed with 0.1% cetyltrimethylammonium bromide, stained with 0.1% toluidine blue in 0.025 M sodium acetate (pH 5.0), and analyzed densitometrically.

To determine the HA molecular weight and concentration of chondroitin sulfate, 1% agarose gels in TAE buffer (0.04 M, pH 8.0) were used. Standards of known molecular weights (rooster comb HA, 800 kDa; bovine trachea HA, 20 kDa) were included. Migration distance was inversely proportional to the log of HA molecular weight. Sulfated glycosaminoglycans were identified by differential toluidine blue staining at different pH values. Hyaluronic acid identification was based on its electrophoretic migration profile and previous validation of the method using selective digestion with testicular hyaluronidase [23,24].

### 2.11. Statistical Analysis

Sample size calculation was performed using G*Power v.3.1.9.4 for an unpaired two-tailed Student’s *t*-test, assuming an effect size of d = 2.0, significance level α = 0.05, and statistical power (1 − β) = 0.95, resulting in a total of 16 animals (8 per group). The effect size was based on previous studies.

Data distribution was assessed using the Shapiro–Wilk test. Parametric variables were analyzed using two-way repeated-measures ANOVA to evaluate the effects of group, time, and their interaction, followed by appropriate post hoc tests. Non-parametric variables were analyzed using Mann–Whitney and Friedman tests. Results are presented as mean ± standard error of the mean for parametric data and median (interquartile range) for non-parametric data.

## 3. Results

### 3.1. Orthopedic Examination

Lameness scores increased significantly at 12 h post-induction in both groups, confirming synovitis. In the HA-treated group, scores progressively decreased after administration and returned to pre-induction values by 48 h, whereas control animals maintained higher scores until 7 d. Significant intergroup differences were observed at 24 and 48 h (Table 1).

Joint effusion returned to pre-induction levels in the treatment group at 24 h, whereas the control group showed resolution only at 48 h.

### 3.2. Ultrasonographic Evaluation

Ultrasonographic evaluation was performed to assess quantitative and qualitative changes in the synovial compartment over time. Among the evaluated ultrasonographic parameters, synovial membrane thickness proved to be the most sensitive and informative variable for detecting treatment-related differences during the acute inflammatory phase.

**Figure 2:**
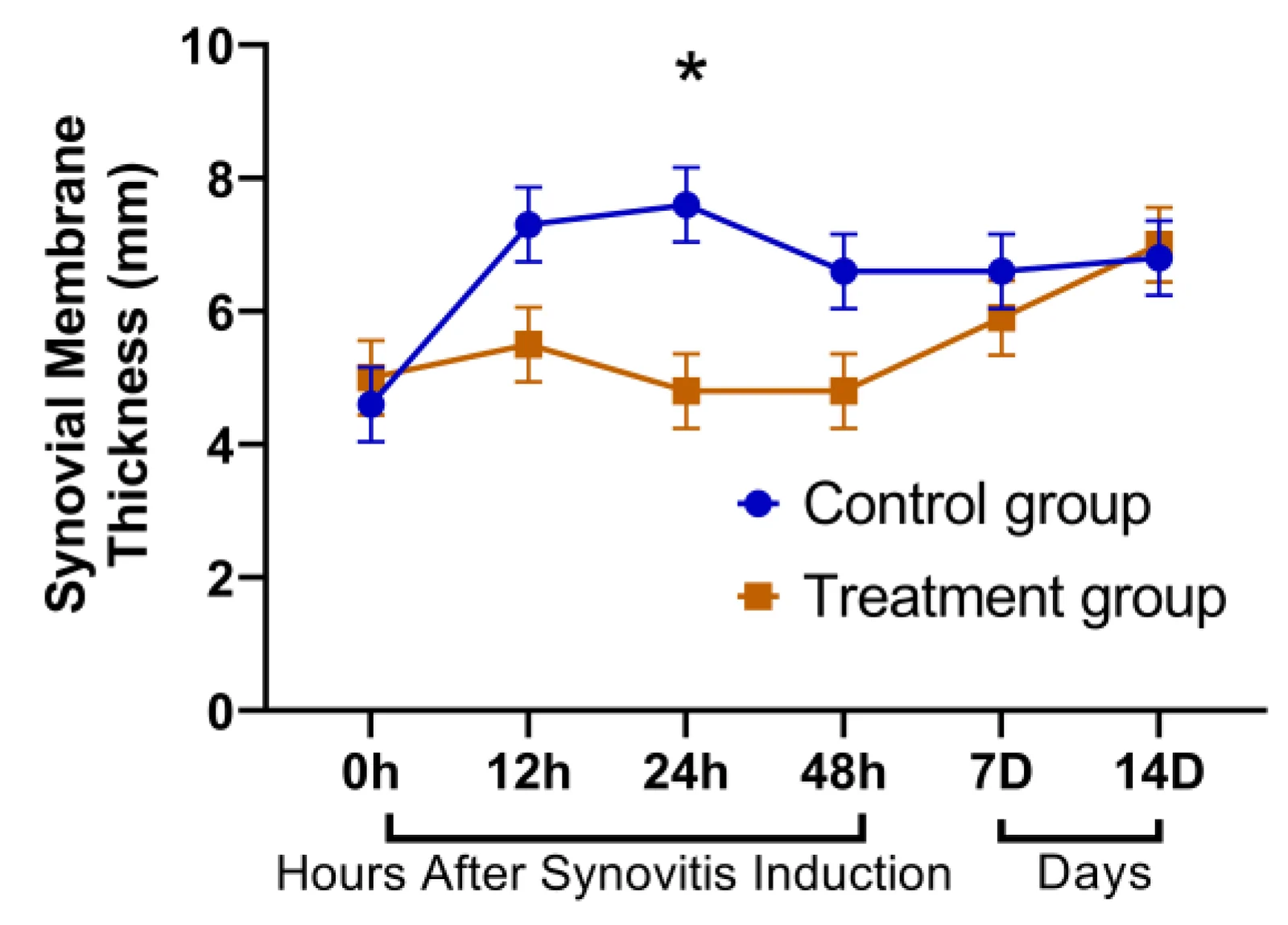
Synovial membrane thickness in control and HMW-HA-treated groups over time. Data are presented as mean ± SEM. Asterisks indicate statistically significant differences between groups at the same time point (* *p* < 0.05). Number signs indicate significant changes within the group over time (*p* < 0.05).

Baseline ultrasonographic examination confirmed joint health in all horses. At 12 h post-induction, before treatment administration, synovial membrane thickness did not differ between groups, indicating a similar degree of synovial response to LPS-induced synovitis. Although a significant increase relative to baseline was observed only within the control group, this was not accompanied by an intergroup difference (Figure 2).

At 24 h, synovial membrane thickness remained close to baseline values in the treatment group, while a further increase was observed in the control group, resulting in a significant intergroup difference. From 48 h onward, no significant differences between groups were detected, although synovial membrane thickness in control joints did not return to baseline values by day 14.

### 3.3. Synovial Fluid Analysis

#### 3.3.1. Cellularity

Synovitis induction caused a significant increase in erythrocytes and neutrophils, confirming inflammation (Table 2). Both cell types returned to baseline by 7 d in both groups, with no intergroup differences at any time point.

#### 3.3.2. Extracellular Matrix Biomarkers

**Figure 3:**
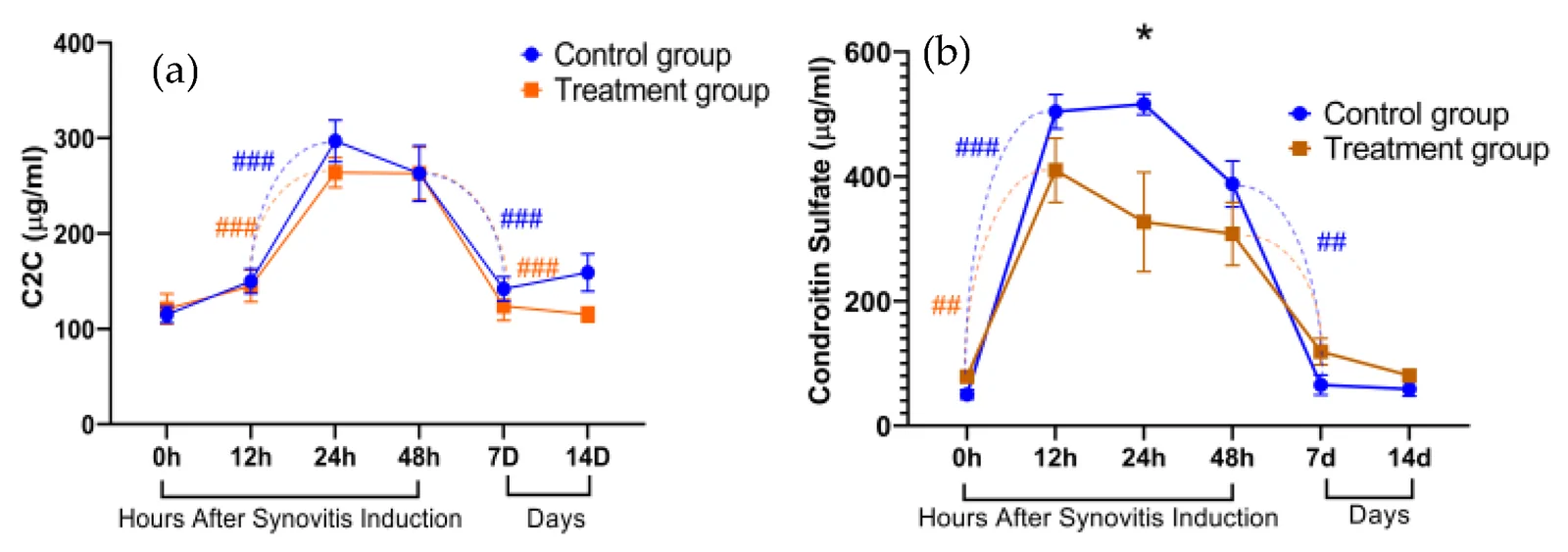
(**a**) Concentration of C2C, a biomarker of type II collagen degradation, in synovial fluid over time. (**b**) Concentration of chondroitin sulfate (CS), a proteoglycan component released during cartilage degradation. Data are expressed as means ± SEM. Asterisks indicate statistically significant differences between groups at the same time point (* *p* < 0.05). Number signs indicate significant changes within-group over time (## *p* < 0.01; ### *p* < 0.001).

C2C levels (type II collagen degradation) increased significantly at 24 h in both groups, then declined to baseline by 7 d (Figure 3a). Chondroitin sulfate (CS) concentrations rose at 12 h in both groups (Figure 3b). At 24 h, CS was significantly higher in controls than in treated horses; this difference disappeared by 48 h, and concentrations normalized by 7 d.

#### 3.3.3. Lipid Peroxidation

**Figure 4:**
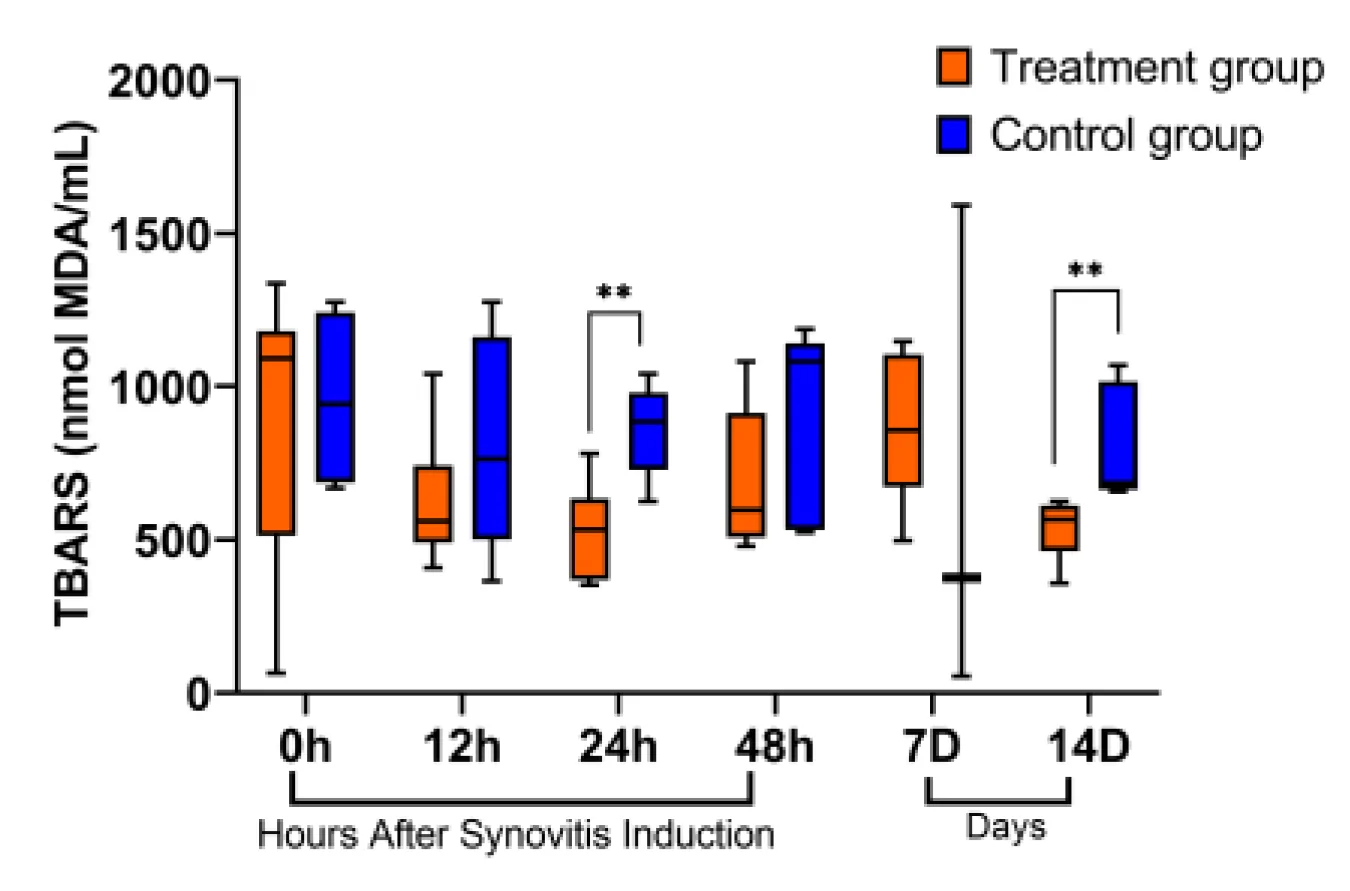
Synovial fluid lipid peroxidation assessed by TBARS following induction of acute synovitis and intra-articular treatment with HMW-HA or PBS. Lower TBARS concentrations were observed in the treated group at 24 h and 14 d. Box plots represent median, interquartile range, and minimum–maximum values. Asterisks indicate statistically significant differences between groups at the same time point (** *p* < 0.01).

TBARS concentration in the synovial fluid differed significantly between groups at two experimental time points: 24 h after synovitis induction (12 h after treatment) and the end of the experiment (14 D), with lower values observed in the treated group (Figure 4).

#### 3.3.4. Hyaluronic Acid Electrophoresis

**Figure 5:**
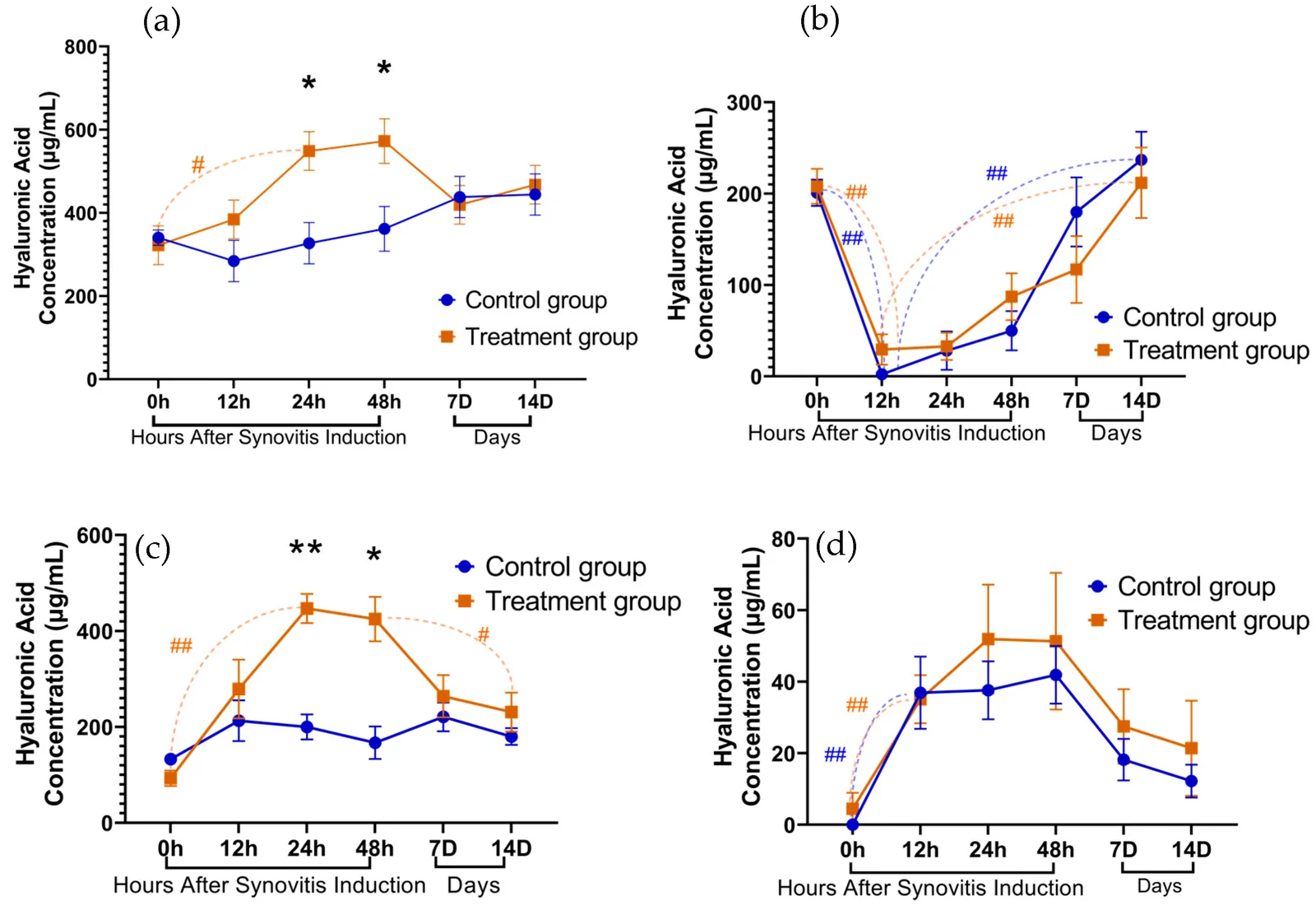
(**a**) Total HA concentration. (**b**) Physiological molecular weight of HA. (**c**) HMW-HA. (**d**) LMW-HA. Data are presented as the means ± SEM at multiple time points. Asterisks indicate statistically significant differences between groups at the same time point (* *p* < 0.05; ** *p* < 0.01). Number signs indicate significant within-group changes over time (# *p* < 0.05; ## *p* < 0.01).

Total HA concentration increased significantly in the treatment group after intra-articular administration (24 h), with significantly higher concentrations compared with the control group. Elevated HA levels in the treated group were maintained until 48 h, corresponding to approximately 36 h of persistence within the synovial fluid (Figure 5a).

Analysis of HA fractions according to molecular weight distribution demonstrated a marked reduction in the physiological HA fraction (6000–20,000 kDa) following synovitis induction, with recovery toward baseline values observed only at day 7 (Figure 5b). The high-molecular-weight HA fraction, corresponding to the administered product, showed a significant increase only in the treated group after administration (24 h). Significant differences between groups were also observed, with treated joints maintaining higher HMW-HA concentrations until 48 h, returning to near-baseline values only at 14 D (Figure 5c). In contrast, the low-molecular-weight HA fraction, which was nearly undetectable at baseline (0 h), increased markedly after synovitis induction in both groups and remained elevated within the synovial environment until the end of the experimental period (14 D) (Figure 5d).

## 4. Discussion

The intra-articular use of hyaluronic acid has been explored through different mechanistic approaches, including mechanical joint protection provided by chemically cross-linked formulations and biological modulation mediated by linear molecules through cellular signaling [11,25]. Following an inflammatory stimulus, proinflammatory cytokines are released, and native hyaluronic acid (HA) molecules are fragmented into low-molecular-weight (LMW) forms that exacerbate inflammation by interacting with receptors such as CD44 and Toll-like receptors 2 and 4, creating a self-perpetuating inflammatory cycle [26,27,28]. In this context, the use of high-molecular-weight (HMW) linear hyaluronic acid aims to modulate this response by preferentially engaging anti-inflammatory pathways, thereby contributing to stabilization of the articular environment.

In the present study, intra-articular lipopolysaccharide (LPS) administration effectively induced acute synovitis and resulted in extensive degradation of endogenous physiological HA, with LMW fragments detected as early as 12 h post-induction and persisting until day 14 in both groups. Physiological HA fractions showed evidence of recovery only by day 7, indicating that the early inflammatory phase was characterized by marked disruption of the synovial HA molecular profile. Low-molecular-weight HA is known to amplify inflammation by promoting cytokine release and contributing to cartilage degradation, reinforcing its role as an endogenous danger signal during synovitis [1].

Following intra-articular administration, HMW-HA was detectable in both groups; however, its concentration was significantly higher in treated joints, with elevated levels maintained between 24 and 48 h post-injection, corresponding to a minimum documented persistence of 36 h based on the sampling intervals evaluated. This quantitative difference indicates that exogenous HMW-HA contributed substantially to the high-molecular-weight HA fraction present during the acute inflammatory phase. Similar transient increases in intra-articular HA concentration following intra-articular administration of linear HA have been reported by Neuenschwander et al. [18], although with shorter duration, likely reflecting differences in formulation, dose, or inflammatory context.

Clinically, lameness scores—both at trot and after flexion—were lower in the treatment group compared with controls, with differences observed from 24 h post-treatment and maintained until day 7, when both groups gradually returned toward baseline values. Improvement in locomotor function occurred concomitantly with increased synovial HMW-HA concentration and preservation of its molecular weight profile, indicating a temporal association between the availability of high-molecular-weight linear hyaluronic acid and reduction in pain-related clinical signs during acute synovitis. In an equine experimental model of LPS-induced synovitis, Neuenschwander et al. [18] similarly reported reductions in lameness following intra-articular administration of linear hyaluronic acid, supporting the relevance of HA-mediated modulation of the synovial environment for clinically detectable locomotor outcomes.

The analgesic potential of hyaluronic acid has been demonstrated in experimental models of joint pain. Gotoh et al. [29] showed that joint nociception is modulated in a molecular-weight-dependent manner, with high-molecular-weight linear HA producing more pronounced and sustained reductions in pain-related walking behavior compared with lower-molecular-weight formulations. Complementarily, Ferrari et al. [30] demonstrated that LMW-HA fragments may induce hyperalgesia through CD44-mediated signaling, whereas HMW-HA exerts analgesic effects, particularly in inflamed tissues. Collectively, these studies reinforce the importance of HA molecular size as a determinant of its nociceptive and clinical effects within the joint.

Ultrasonographic evaluation revealed increased synovial membrane thickness in control joints from 12 h post-induction, persisting until day 14. In contrast, treated joints maintained synovial membrane thickness close to baseline values and exhibited significantly lower synovial membrane thickness at 24 h. These findings suggest attenuation of synovial tissue response associated with acute inflammation following intra-articular administration of HMW-HA [31,32,33].

Alterations in synovial fluid composition during synovitis—including elevations in proinflammatory cytokines (e.g., IL-1β and IL-6), tumor necrosis factor, matrix metalloproteinases, hyaluronidases, and nitric oxide—contribute to cartilage degradation and impair the synthesis of protective macromolecules such as HMW-HA [12,13,20,31,32]. This biochemical environment perpetuates inflammation and disrupts joint homeostasis. Within this context, exogenous administration of HMW-HA has been proposed to support restoration of articular balance by stimulating resident joint cells to produce HA, glycosaminoglycans, and collagen [3,7,9,17,34]. Although inflammatory mediators were not directly quantified in the present study, the associations observed between HMW-HA administration, preservation of HA molecular stability, and improvement of clinical, ultrasonographic, and biochemical parameters are consistent with this proposed framework.

Synovitis induction resulted in a robust inflammatory response within the synovial fluid, characterized by increased neutrophil and mononuclear cell counts and elevated plasma protein concentrations, consistent with previously reported LPS-induced equine models [19,20]. Importantly, the absence of significant differences in leukocyte counts between treated and control joints indicates that inflammatory cell recruitment occurred similarly in both conditions, suggesting that the observed effects associated with HMW-HA administration were not driven by quantitative changes in cellular infiltration. This observation supports the hypothesis that linear HMW-HA may exert its modulatory effects through qualitative changes in inflammatory cell function rather than changes in cell number.

In this context, Lee et al. [35,36] demonstrated that HMW-HA promotes alternative macrophage activation pathways and shifts classically activated macrophages toward an anti-inflammatory phenotype. Although macrophage polarization was not directly evaluated in the present study, these findings provide a biologically plausible explanation for improved outcomes despite comparable leukocyte counts and highlight macrophage-centered mechanisms as a relevant focus for future in vivo investigations.

Lipid peroxidation assessed by the TBARS assay revealed two distinct experimental time points with differences between groups, at 24 h after synovitis induction and at 14 days, in which the treated group showed lower concentrations compared with the control group. This method reflects lipid peroxidation and was therefore used to assess this process within the synovial environment.

At 24 h, the administration of HMW-HA was associated with lower lipid peroxidation levels, suggesting modulation of the redox environment. At 14 days, even in the absence of alterations in other evaluated parameters or signs of active inflammation, the treated group maintained lower TBARS levels.

Oxidative stress occurs when there is an imbalance between the production and neutralization of reactive oxygen and nitrogen species [15,16]. In the present study, some findings may be related to this context, including the increase in leukocytes observed during the acute phase, structural alterations that may contribute to changes in the joint microenvironment, and possible effects on cellular metabolism.

In addition to inflammatory cell recruitment, functional changes in these cells may also be relevant. In this regard, the functional modulation of inflammatory cells, particularly macrophages, may influence the production of reactive species independently of cell number [36,37,38,39]. In this context, the structural findings observed in the ultrasonographic evaluation and the preservation of the synovial membrane in the treated group suggest a more stable articular environment, which may also be associated with preservation of cellular metabolism and mitochondrial function [33].

Thus, the findings of the present study do not necessarily indicate prevention of oxidative damage under the evaluated conditions but suggest that HMW-HA may contribute to maintaining a more balanced redox state within the joint environment.

Although the present findings support a biological modulatory effect of HMW-HA within the synovial environment, some mechanisms were not directly investigated. Inflammatory cytokines, macrophage activation, and polarization were not evaluated, limiting mechanistic interpretation of the observed responses. In addition, oxidative status was assessed only through TBARS determination, and additional biomarkers could provide a more comprehensive characterization of redox alterations. Furthermore, although sample size calculation was based on previous studies employing similar experimental models, subtle biological effects may not have been detected. Further studies, including broader molecular evaluations and additional sampling time points, may help clarify the temporal dynamics and biological mechanisms associated with HMW-HA administration.

Taken together, the present findings demonstrate that intra-articular administration of high-molecular-weight linear hyaluronic acid modulates the synovial environment during acute synovitis by increasing the availability of HMW-HA, attenuating synovial tissue response, and improving pain-related clinical parameters without affecting inflammatory cell recruitment. In addition to these effects, the differences observed in lipid peroxidation suggest that this modulation may extend to the redox balance within the joint. Although no intragroup changes in TBARS levels were detected over time, the consistently lower values observed in the treated group indicate a more balanced articular microenvironment. This finding supports the concept that HMW-HA may influence not only inflammatory pathways but also the metabolic and oxidative status of the joint environment.

Overall, these results reinforce the hypothesis that linear HMW-HA acts primarily through biological modulation of the intra-articular milieu, contributing to the maintenance of joint homeostasis during and after the acute inflammatory phase.

## 5. Conclusions

In this equine model of acute synovitis, synovial inflammation resulted in marked alterations in hyaluronic acid molecular profile, characterized by degradation of endogenous high-molecular-weight fractions. Intra-articular administration of high-molecular-weight linear hyaluronic acid increased the availability of HMW-HA within the joint during the acute inflammatory phase.

This increase was associated with reduced lameness scores, lower synovial membrane thickening, and lower release of extracellular matrix components. Additionally, treated joints exhibited lower lipid peroxidation levels, suggesting a more balanced redox environment.

Collectively, these findings support a modulatory role of linear HMW-HA in the intra-articular environment, contributing to the preservation of joint homeostasis through effects on structural, clinical, and metabolic parameters during acute synovitis.

## Figures and Tables

**Table 1:** Orthopedic assessment: lameness and joint effusion scores over time.

Parameter		0 h	12 h	24 h	48 h	7 d	14 d
Lameness at walk (0–5)	Control	0 (0–0) ^a,A^	2.5 (2–3) ^a,B^	2 (1–2.7) ^b,B^	1.5 (1–2) ^b,B^	0 (0–0.7) ^a,A^	0 (0–0) ^a,A^
	Treatment	0 (0–0) ^a,A^	1 (0–2.7) ^a,B^	0 (0–1.7) ^a,A^	0 (0–0.7) ^a,A^	0 (0–0.7) ^a,A^	0 (0–0) ^a A^
*p* value		>0.999	0.075	0.029	0.0047	>0.999	>0.999
Lameness at trot (0–5)	Control	0 (0–0) ^a,A^	2.5 (2–3) ^a,B^	2 (1.2–2.7) ^b,B^	2 (1–2) ^b,B^	0 (0–0.7) ^a,A^	0 (0–0.7) ^a,A^
	Treatment	0 (0–0) ^a,A^	2 (1–2.7) ^a,B^	1 (0–1) ^a,A^	1 (0–1) ^a,A^	0 (0–0) ^a,A^	0 (0–0) ^a,A^
*p* value		>0.999	0.327	0.013	0.008	0.466	0.466
Lameness postflexion (0–5)	Control	0 (0–0) ^a,A^	2.5 (2–3) ^a,B^	2 (2–3) ^b,B^	2 (2–2) ^b,B^	0 (0–1.5) ^a,A^	0 (0–1.7) ^a,A^
	Treatment	0 (0–0) ^a,A^	2 (1.2–2.7) ^a,B^	1 (0.2–1.7) ^a,A^	1 (0–1) ^a,A^	0 (0–0.7) ^a,A^	0 (0–0) ^a,A^
*p* value		>0.999	0.274	0.003	<0.001	0.712	0.323
Joint effusion (0–4)	Control	0 (0–0) ^a,A^	1 (1–1.7) ^a,B^	1 (1–2) ^a,B^	1 (0.2–1.7) ^a,A^	0 (0–1) ^a,A^	0 (0–0) ^a,A^
	Treatment	0 (0–0) ^a,A^	2 (1–2.7) ^a,B^	1 (1–1) ^a,A^	1 (0–1) ^a,A^	0 (0–0.7) ^a,A^	0 (0–0) ^a,A^
*p* value		>0.999	0.345	0.592	0.689	>0.999	>0.999

Data are presented as median (interquartile range). Different lowercase letters (a, b) within the same column indicate significant differences between groups at a given time point. Different uppercase letters (A, B) within the same row indicate significant differences over time within the same treatment group (*p* < 0.05).

**Table 2:** Synovial fluid cellularity in control and treatment groups over time.

Cell		0 h	12 h	24 h	48 h	7 d	14 d
Erythrocytes (log scale)	Control	1.9 ± 0.4 ^A^	5.4 ± 0.4 ^B^	5.5 ± 0.4 ^B^	5.0 ± 0.4 ^B^	2.1 ± 0.4 ^A^	2.0 ± 0.4 ^A^
	Treatment	2.0 ± 0.4 ^A^	4.7 ± 0.4 ^B^	5.0 ± 0.4 ^B^	5.3 ± 0.4 ^B^	3.7 ± 0.4 ^A^	3.0 ± 0.4 ^A^
*p* value		>0.999	0.996	0.999	>0.999	0.201	0.855
Neutrophils (log scale)	Control	0.7 ± 0.1 ^A^	4.5 ± 0.1 ^B^	4.1 ± 0.1 ^B^	3.5 ± 0.1 ^B^	1.5 ± 0.1 ^A^	1.4 ± 0.1 ^A^
	Treatment	0.4 ± 0.1 ^A^	4.1 ± 0.1 ^B^	3.7 ± 0.1 ^B^	3.3 ± 0.1 ^B^	0.9 ± 0.1 ^A^	1.2 ± 0.1 ^A^
*p* value		0.984	0.894	0.878	>0.999	0.486	0.998
Mononuclear cells (cells/μL)	Control	156 ± 76.3 ^A^	4593 ± 788 ^A^	4617 ± 1199 ^B^	2738 ± 576 ^A^	372 ± 22 ^A^	288 ± 40.3 ^A^
	Treatment	204 ± 180 ^A^	4881 ± 2393 ^A^	2690 ± 283 ^A^	2917 ± 695 ^B^	624 ± 298 ^A^	220 ± 28.2 ^A^
*p* value		>0.999	>0.999	0.897	>0.999	0.999	0.951

Synovial fluid cellularity in control and HMW-HA-treated groups over time. Data are expressed as mean ± SEM. Different uppercase letters (A, B) in the same row indicate significant differences over time within the same group (*p* < 0.05).

## Data Availability

https://doi.org/10.6084/m9.figshare.30074989 (accessed on 14 July 2026).
